# Phylotranscriptomics suggests the jawed vertebrate ancestor could generate diverse helper and regulatory T cell subsets

**DOI:** 10.1186/s12862-018-1290-2

**Published:** 2018-11-15

**Authors:** Anthony K. Redmond, Daniel J. Macqueen, Helen Dooley

**Affiliations:** 1 School of Biological Sciences, University of Aberdeen, Aberdeen, AB24 2TZ UK; 2 Centre for Genome-Enabled Biology & Medicine, University of Aberdeen, Aberdeen, AB24 2TZ UK; 3 Department of Microbiology & Immunology, University of Maryland School of Medicine, Institute of Marine & Environmental Technology, 701 E Pratt St, Baltimore, MD21202 USA; 4 Present address: Smurfit Institute of Genetics, Trinity College Dublin, University of Dublin, Dublin 2, Ireland

**Keywords:** Cartilaginous fish, Elasmobranch, Shark, Immunity, T cell, Interleukin, Immune gene evolution, Site-heterogeneous models, Phylogenetic rooting, Transcriptome

## Abstract

**Background:**

The cartilaginous fishes diverged from other jawed vertebrates ~ 450 million years ago (mya). Despite this key evolutionary position, the only high-quality cartilaginous fish genome available is for the elephant shark (*Callorhinchus milii*), a chimaera whose ancestors split from the elasmobranch lineage ~ 420 mya. Initial analysis of this resource led to proposals that key components of the cartilaginous fish adaptive immune system, most notably their array of T cell subsets, was primitive compared to mammals. This proposal is at odds with the robust, antigen-specific antibody responses reported in elasmobranchs following immunization. To explore this discrepancy, we generated a multi-tissue transcriptome for small-spotted catshark (*Scyliorhinus canicula*), a tractable elasmobranch model for functional studies. We searched this, and other newly available sequence datasets, for CD4+ T cell subset-defining genes, aiming to confirm the presence or absence of each subset in cartilaginous fishes.

**Results:**

We generated a new transcriptome based on a normalised, multi-tissue RNA pool, aiming to maximise representation of tissue-specific and lowly expressed genes. We utilized multiple transcriptomic datasets and assembly variants in phylogenetic reconstructions to unambiguously identify several T cell subset-specific molecules in cartilaginous fishes for the first time, including interleukins, interleukin receptors, and key transcription factors. Our results reveal the inability of standard phylogenetic reconstruction approaches to capture the site-specific evolutionary processes of fast-evolving immune genes but show that site-heterogeneous mixture models can adequately do so.

**Conclusions:**

Our analyses reveal that cartilaginous fishes are capable of producing a range of CD4+ T cell subsets comparable to that of mammals. Further, that the key molecules required for the differentiation and functioning of these subsets existed in the jawed vertebrate ancestor. Additionally, we highlight the importance of considering phylogenetic diversity and, where possible, utilizing multiple datasets for individual species, to accurately infer gene presence or absence at higher taxonomic levels.

## Background

The cartilaginous fishes (Chondrichthyes) diverged from a common ancestor with other vertebrates around 450 million years ago (mya) and are comprised of Holocephali (chimaeras) and Elasmobranchii (sharks, skates, and rays), which likely split between 300 and 420 mya [1, 2]. They represent the most phylogenetically-distant relatives of mammals to have an adaptive immune system based on somatically-rearranging immunoglobulins (i.e. antibodies) and T cell receptors, as well as major histocompatibility complex molecules [3, 4]. Despite their key evolutionary position, the only high-quality genome assembly available for this group is that of the elephant shark (*Callorhinchus milii*); a chimaera [5]. This dataset has been used to infer the presence or absence of many genes in the cartilaginous fishes [5]. However, distinct scenarios of gene family evolution are likely to have played out within cartilaginous fish evolutionary history, most notably across the vast time separating chimaeras and elasmobranches (e.g [6]), questioning the use of a single species to infer the presence or absence of genes in an entire vertebrate class.

**Fig. 1:**
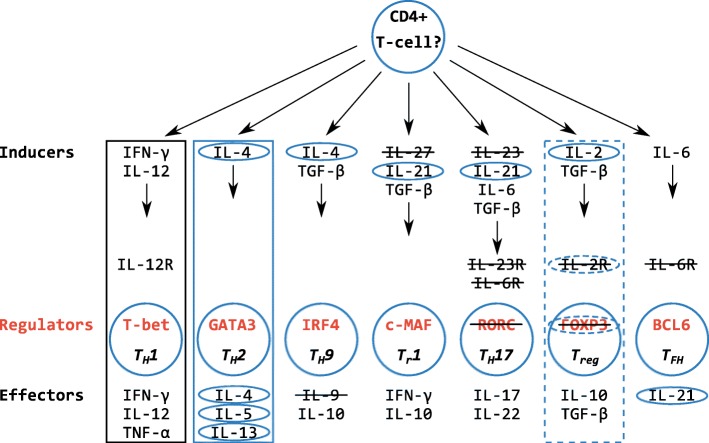
Summary of the presence/absence of major mammalian CD4+ T-cell lineages and associated genes in the jawed vertebrate ancestor. The figure and gene selection are based on Fig. 5 from Venkatesh et al. [5]. Boxed lineages were predicted to have emerged in the ancestor of jawed vertebrates by Venkatesh et al. [5] (black boxed lineages), or by Dijkstra [18] (blue boxed lineages). Crossed out genes are those thought to be absent from cartilaginous fishes and the vertebrate ancestor, while blue encircled genes are those that Dijkstra later predicted to in fact be present [18]. Dotted box/circle edges indicate uncertainty of gene or lineage presence(e.g. for IL-2R, FOXP3 [18])

In this respect, an initial survey of the elephant shark genome suggested the immune gene repertoire of cartilaginous fishes was very different to that of bony jawed vertebrates, lacking many CD4+ T cell-associated genes present in mammals [5]. T cells expressing the CD4 co-receptor are vital for mounting an adaptive immune response [7], and are loosely split into two major groups: (i) so-called ‘helper’ T cells, required for antibody production and subsequent affinity maturation of the response, in addition to immunological memory [7–11], and (ii) ‘regulatory’ T cells that suppress immune responses, preventing autoimmunity [12, 13]. These two groups are further divided into functionally-specific subsets (i.e. T_H_1, T_H_2, T_H_9, T_H_17, T follicular helper [T_FH_], T_reg_, and T_r_1 cells), dependent upon on the cytokines (soluble signalling molecules such as interleukins [ILs]) and transcription factors required for their development, combined with the cell surface receptors and effector cytokines they express (Fig. 1) [7–9, 11–17]. The apparent absence of many of these molecules from elephant shark led to a proposal that cartilaginous fishes possess only a primordial T helper (T_H_) cell system, based upon the T_H_1 subset alone [5]. Subsequent analyses [18] suggested genes associated with the T_H_2 and T_reg_ subsets may be present, although in some cases the evidence was weak [19]. The proposal that cartilaginous fishes lack a diverse set of T cell subtypes is at odds with evidence that elasmobranchs can generate a robust antibody response, incorporating immunological memory and affinity maturation, following immunization [20, 21].

To robustly infer orthology, as well as gene duplication and loss events, when comparing cartilaginous fish and their sister bony vertebrates, highly complete datasets are required. Adding to the elephant shark genome data, draft genome sequences have also become available for little skate (*Leucoraja erinacea*) [6, 22, 23] and whale shark (*Rhincodon typus*) [24]. While extremely useful tools for comparative analyses, draft assemblies may often be limited by their fragmented nature, a problem compounded by the large and complex genomes of elasmobranches [25, 26]. Several cartilaginous fish transcriptomes are also available [6, 27–36], but none include a complete set of immune tissues. For these reasons, one goal of the current study was to generate elasmobranch transcriptome data that includes a full set of immune tissues, in the hope of revealing immune genes missed by other studies.

Even with high-quality data at hand, characterising the evolution of immune gene families across deep timescales remains challenging. The divergence of cartilaginous and bony jawed vertebrates occurred a relatively short time after genome duplication event(s) [37–40] that radically re-shaped the evolution of many immune gene families [41, 42]. This could result in limited or conflicting phylogenetic signals, especially for rapidly-evolving genes. Robust phylogenetic inference of immune gene families at deep evolutionary scales should therefore include measures to avoid poorly fitting models of amino acid substitution and the inclusion of inappropriate outgroups [43–45]. Mixture models accounting for variation in the amino acid substitution process across sites (i.e. site-heterogeneous models), such as CAT [46], can improve phylogenetic inference at deep evolutionary timescales when compared to site-homogeneous models [44] and have been adapted for smaller datasets [47, 48]. We hypothesize that this property will transfer particularly well to immune genes, due to the presence of rapid evolutionary turnover at some amino acid positions, in a background of structural conservation.

The principal aim of this study was to robustly reconstruct the T cell subset diversity of cartilaginous fishes in the context of jawed vertebrate evolution. To achieve this aim, we first generated a normalised multi-tissue transcriptome dataset for small-spotted catshark, a model cartilaginous fish species [49]. Our approach aimed to produce a ‘genome proxy’ maximising representation of novel genes expressed from immune tissues. Second, we searched for homologues of gene families involved in CD4+ T cell development and function in multiple catshark transcriptome datasets, along with genome and transcriptome data from other species of cartilaginous fish. Third, we used these sequences for phylogenetic reconstructions employing relaxed clock and outgroup rooting approaches, and considering the relative and absolute fit of site-heterogeneous mixture models. The results allowed us, for the first time, to unambiguously identify several CD4+ T cell-associated genes in cartilaginous fishes, implying that the ancestral jawed vertebrate was, and cartilaginous fishes are, capable of producing an array of helper and regulatory T cell subsets comparable to that of modern mammals. We also provide important insights into the phylogenetic analysis of fast-evolving immune genes at deep evolutionary timescales.

## Materials & methods

### Sample preparation and sequencing

A captive-bred, female small-spotted catshark of approximately 3 years of age was overdosed with MS-222 prior to sacrifice and tissue harvest; all procedures were conducted in accordance with UK Home Office ‘Animals and Scientific Procedures Act 1986; Amendment Regulations 2012’ on animal care and use, with prior ethical approval from the University of Aberdeen’s Animal Welfare and Ethical Review Body (AWERB). Tissue samples were flash frozen at − 80 °C for stomach, liver, spleen, gill, and brain, as well as spiral valve (homologous to the large intestine), and the epigonal and Leydig organs (associated with the gonad and oesophagus, respectively; bone marrow equivalents of cartilaginous fishes: [50]). Total RNA isolation was performed using TRIzol (Sigma-Aldrich) according to the manufacturer’s instructions but washing the RNA pellets 4x with 70% ethanol. Homogenisation was performed using either mortar and pestle, or tungsten carbide beads (QIAGEN) and a TissueLyser II (QIAGEN). RNA Quantification was performed by broad range RNA assay on a Qubit 2.0 fluorimeters (Invitrogen). RNA integrity was assessed via the Agilent 2100 Bioanalyzer or 2200 TapeStation (Agilent technologies) platforms. RNA samples were pooled to create a single multi-tissue RNA sample which was used for subsequent cDNA synthesis and normalisation using the Evrogen Mint-2 and Trimmer-2 kits, respectively, performed according to manufacturer’s instructions. cDNA (both normalised and non-normalised) was quantified by Qubit dsDNA HS Assay, and quality assessed by TapeStation. An additional peak at 166 bp, only present in the normalised sample, and probably representing a primer dimer introduced in the normalisation process, was removed by AMPure XP clean-up and verified on the TapeStation.

The sequencing library was constructed with the Ion Xpress Plus gDNA Fragment Library preparation kit (Life Technologies). Size selection was performed using a BluePippen (Sage Science) with a 270 bp target size and confirmed via TapeStation. The Ion Library quantification kit (Life Technologies) was used to quantify the library by qPCR on an Illumina ECO qPCR machine, revealing the need for amplification, which was performed according to the library preparation protocol. AMPure XP clean-up of the amplified library was then performed along with final TapeStation quality assessment and quantification via qPCR. Sequencing was performed on the Ion Proton (Life Technologies) using 2x Ion PI v2 BC Chips (Life Technologies) to generate single-ended 200 bp reads. The Ion PI IC 200 kit (Life Technologies) was used, and chips were prepared by the Ion Chef (Life Technologies).

### Transcriptome assembly

The quality of each dataset was examined using fastqc (v0.10.1) [51]. Adaptors were trimmed using fastq-mcf within the ea-utils package (v1.1.2) [52], along with low quality bases from the start and end of reads, and very short sequences (named ‘MCF’ dataset after the trimming protocol). A hard-trimming procedure was also performed using a custom Perl script, where the first 10 bases, and any bases after 250 bp were trimmed from every read (named ‘HTMCF’ dataset; ‘HT’ representing ‘hard trim’ in addition to the MCF procedure), given that reads of greater than this length are most likely spurious. De novo assemblies were performed in Trinity (r2013-11-10) [53] for both datasets, as well as for an untrimmed dataset (named ‘RAW’ dataset). Assemblies were evaluated using basic assembly metrics for transcripts > 300 bp using the Assemblathon2 Perl script Assemblathon_Stats.pl (downloaded from http://korflab.ucdavis.edu/datasets/Assemblathon/Assemblathon2/Basic_metrics/assemblathon_stats.pl) [54]. BUSCO (v 3.0.0) analyses were performed against vertebrate and metazoan datasets (odb9), with no transcript size cut-off [55].

### Sequence searches and phylogenetic analyses

Characterized human, chicken, teleost, or putative elephant shark amino acid sequences (as identified by [18]) for CD4+ T cell-associated genes identified as ‘missing’ from cartilaginous fishes, were used as TBLASTN [56] queries against each of the three transcriptome assemblies (RAW, MCF, HTMCF), as well as against two existing small-spotted catshark transcriptomes: ‘MEA’, from Mulley et al. [30], and ‘KEA’, from King et al. [6] (e-value cut-off: 10). In addition to using multiple catshark datasets, searches were also performed against the recently released spiny dogfish (*Squalus acanthias*, a member of the distantly related Squaleomorphii) [57] and blue shark (*Prionace glauca*, as this included spleen) transcriptomes [36], and the recently improved whale shark genome assembly (*Rhincodon typus*; ASM164234v2) [24]. The top 10 hits for each search were translated using TransDecoder v2.1 [58] and used as BLASTP queries against the Swissprot reviewed database. Those sequences producing hits to the protein of interest or a close relative were retained for phylogenetic analysis. To complement the BLAST analyses, profile hidden Markov model (HMM) based searches were also applied in HMMER v3.1 [59], using an alignment of phylogenetically diverse and representative orthologues as a query. All HMMER analyses were performed against either predicted protein models for whale shark, or TransDecoder translated transcriptomes for other species. All hits above the default exclusion threshold in HMMER were extracted and used as BLASTP queries against Swissprot to detect homologs to sequences of interest.

The newly identified sequences were mainly interleukins (IL), which are cytokines (cell signalling molecules) of the immune system, and their receptors (IL-R), along with potential T_H_17 and T_reg_ transcription factors. To verify the identity of hits by phylogenetic analysis, as well as to assess evidence for loss of other CD4+ T cell-associated genes, nine datasets were assembled. For IL-Rs three datasets were generated; an IL-6Rα family dataset based on [60], an IL-2Rα/IL-15Rα dataset, and a class 1 group 2 cytokine receptor family dataset, as defined by [61]. Two main datasets were generated for ILs; an IL-6 superfamily dataset with members of both the IL-6 and IL-12 families included (as some genes appear to co-occupy these families [62–64]), and an IL-2 superfamily dataset. The IL-2 family dataset was further broken down into more focused subsets, one containing IL7 and IL9 (which are considered sister genes [65, 66]), including the closely related IL-2 superfamily members IL-4 and IL-13 as outgroups [66], and a second focusing on the aforementioned IL-4 and IL-13, using IL2, IL-15, and IL-21 as an outgroup [66]. Finally, two transcription factor family datasets were also assembled. A dataset of the retinoic acid receptor-related orphan receptor (ROR) transcription factor family was compiled, considering two other nuclear receptors as outgroups; the human retinoic acid receptors (RARs), as well as nuclear hormone receptor 3 (HR3) of fruit fly [67]. A dataset for the forkhead-box P (FOXP) family was also assembled, with the use of invertebrate FOXP sequences as the outgroup tested.

Multiple sequence alignments were generated using MAFFT v7 [68], and trimmed using the ‘gappyout’ approach in trimAl v1.2 [69]. Maximum likelihood phylogenetic analyses were performed in IQ-tree (omp-1.5.4) with 1000 ultrafast bootstrap replicates [70, 71]. Model selection was carried out in IQ-tree under the Bayesian information criterion (BIC), mainly considering the standard substitution models available in BEAST [72] and Phylobayes [73]. The fit of the phylogenetic mixture models C10, C20, C30, C40, C50 and C60 (empirical CAT models) [47] were also examined, as well as variants with ‘+F’, ‘+JTT/WAG/LG’, or ‘+JTT/WAG/LG + F’ for the empirical CAT models (‘+G’ is already included in IQ-tree). These combinations were applied as the CAT model has been shown to provide a better fit to many datasets when combined with GTR (yielding GTR-CAT) [43, 74–76], and JTT/WAG/LG + C10/C20/30/40/50/60 might be viewed as providing a precomputed GTR-CAT mimic (see also: [77]).

Bayesian phylogenetics in Phylobayes 4.1b [73] were performed in cases where mixture models were better fitting in the IQ-tree analysis, but using the CAT model itself rather than the empirical derivation (for example, JTT + C10 and C40 would be replaced by JTT + CAT and CAT, respectively), as this, theoretically, should collapse to the most appropriate number of site categories, is commonly used in phylogenomics, and has been shown to perform well for gene family analyses elsewhere [78, 79]. While the CAT model has been applied in previous studies of immune genes [80, 81], its fit to such datasets has never been tested. As such, in addition to testing the relative fit of empirical CAT models in IQ-tree, posterior predictive simulations (PPS) [44] were performed in Phylobayes to test if CAT-based models offer an improved absolute fit (in terms of describing site-specific amino acid alphabets), over standard models for fast-evolving immune genes. A standard statistical cut-off for a two-sided test was applied (at *P* < 0.05), such that posterior predictive Z-scores > 1.96 or < − 1.96 significantly deviate from the observed value (i.e. from the real data) and the model is taken to be inadequate.

Bayesian phylogenetics incorporating an outgroup-free relaxed clock rooting approach, which we have previously applied successfully to root vertebrate immune gene family trees [82–86], were performed in BEAST v1.8.3 [72], using an uncorrelated relaxed clock model [87], and a Yule speciation prior [88, 89]. Two Markov chain Monte Carlo samples were generated in all Bayesian analyses, with convergence of sampled chains assessed in Phylobayes as maxdiff < 0.3, and visually appraised in Tracer v1.6 (http://beast.bio.ed.ac.uk/tracer) for BEAST runs. To summarise these analyses, 50% majority rule consensus trees were generated for Phylobayes runs, while RootAnnotator [90] was used to obtain root probabilities and identify a maximum clade credibility tree from the BEAST Markov chain Monte Carlo sample.

## Results

### Small-spotted catshark transcriptome

Ion Proton sequencing returned 134,841,605 reads, from which three Trinity assemblies based on increasingly complex quality filtering strategies were generated: RAW, MCF, and HTMCF, respectively containing 623,430, 621,635, and 167,255 contigs (See Table 1 for extended statistics for contigs > 300 bp). Compared to the MCF and RAW datasets, the HTMCF dataset produced a reduced number of contigs (Table 1), a greater number of complete single-copy BUSCOs (Table 2), and a higher N50 contig length (Table 1), suggesting that more stringent read and base quality filtering improves assembly contiguity. However, the RAW and MCF assemblies contain fewer missing BUSCOs, suggesting that novel data was lost from the HTMCF assembly (Table 2).

**Table 1:** Basic assembly statistics (> 300 bp) compared to existing small-spotted catshark transcriptomes

	KEA	MEA	RAW	MCF	HTMCF
Total base count	*45,214,552*	*110,464,397*	275,457,098	275,410,367	80,066,015
Total contig count	58,273	86,006	388,800	387,579	102,228
N50^a^ contig length	965	2316	786	791	969
L50^b^ contig count	12,721	13,518	89,641	88,973	20,814
GC content (%)	45.69	43.9	42.09	42.1	42.03

^a^N50: the length of the shortest contig included when the least number of contigs are used to make up 50% of the total assembly length

^b^L50: the total contig count when the least number of contigs are used to make up 50% of the total assembly length

**Table 2:** RAW, MCF and HTMCF, BUSCOs compared to existing catshark transcriptomes

	KEA	MEA	RAW	MCF	HTMCF
*Vertebrata* (2586 BUSCOs)					
Complete Single-copy BUSCOs	1392 (53.83%)	1257 (48.61%)	997 (38.55%)	1000 (38.67%)	1533 (59.28%)
Complete Duplicated BUSCOs	164 (6.34%)	788 (30.47%)	434 (16.78%)	435 (16.82%)	188 (7.27%)
Fragmented BUSCOs	641 (24.79%)	277 (10.71%)	852 (32.95%)	855 (33.06%)	477 (18.45%)
Missing BUSCOs	389 (15.04%)	264 (10.21%)	303 (11.72%)	296 (11.45%)	388 (15%)
*Metazoa* (978 BUSCOs)					
Complete Single-copy BUSCOs	735 (75.15%)	628 (64.21%)	583 (59.61%)	588 (60.12%)	787 (80.47%)
Complete Duplicated BUSCOs	87 (8.9%)	300 (30.67%)	248 (25.36%)	238 (24.34%)	75 (7.67%)
Fragmented BUSCOs	116 (11.86%)	33 (3.37%)	127 (12.99%)	130 (13.29%)	78 (7.97%)
Missing BUSCOs	40 (4.09%)	17 (1.74%)	20 (2.04%)	22 (2.25%)	38 (3.89%)

Comparison with existing catshark datasets suggested that the total number of transcripts and bases in our assemblies is greater than generated elsewhere (Table 1). All assemblies lack some BUSCOs, however the MEA assembly contains the fewest missing BUSCOs. Both the MEA (liver, pancreas, and brain [30]) and KEA (pooled embryo [6]) datasets were sequenced on the Illumina platform, raising the possibility that the increased number of transcripts in our assemblies is linked to the increased error rates under Ion Torrent sequencing [91, 92]. In addition, the apparent increased contiguity of the MEA assembly is likely due to the use of paired-end reads, which was not done here. Biological explanations are also possible for the differences, such as the introduction of additional sequences and splice variants from multiple tissues [93], or the inclusion of very lowly expressed transcripts, due to normalisation.

Overall, a combination of sequencing approaches, as well as biological differences are likely to significantly impact the gene content of each assembly. For this reason, all five assemblies were carried forward to allow a robust search for T cell-associated genes in cartilaginous fishes.

### CD4+ T cell subset-defining genes in cartilaginous fishes

To better understand T cell biology in cartilaginous fishes, we employed BLAST and HMM based searches of all five small-spotted catshark assemblies, as well as blue shark and spiny dogfish transcriptomes, and the whale shark predicted proteome, for the ILs and IL-Rs defining those T cell subsets reported missing from elephant shark [5]. These searches failed to identify putative homologs of IL-2 or its receptor IL-2R, IL-5, IL-9, or RORC. However, we identified putative sequences for IL-4/IL-13, IL-21, IL-23, IL-27 (p28), IL-6Rα, IL-23R, and FOXP3. Presence of the IL and IL-R sequences was variable between species, and between catshark transcriptome datasets (Table 3). To verify orthology of these sequences we assembled comprehensive datasets for each gene along with related family members and performed extensive phylogenetic analyses.

**Table 3:** Demonstration of putative orthologue content variation between datasets

	KEA	MEA	RAW	MCF	HTMCF
IL-4/13	✗	✗	✓	✓	✗
IL-27 (p28)	✗	✗	✓	✓	✓
IL-6Rα	✓	✓	✗	✗	✗
IL-23R	✗	✗	✓	✓	✓

### Site heterogeneous mixture models capture site-specific evolutionary processes in immune genes where standard models fail

The relative fit of empirical mixture models was tested for each of the assembled datasets, as these models may better accommodate the complex evolutionary history of fast-evolving genes. Simple empirical CAT models were never better-fitting than standard site-homogeneous models for any dataset according to the BIC (Table 4). However, when these were paired with a standard model (i.e. JTT, WAG, or LG), this resulted in improved fit over standard substitution models for the IL-6 superfamily, class-1 group-2 cytokine receptor, and FOXP datasets.

**Table 4:** IQ-tree BIC best-fit model results for all dataset

Dataset	Best overall	Best standard
*IL-2 superfamily*	JTT + F + G	JTT + F + G
*IL-7/9 family no outgroup*	JTT + G	JTT + G
*IL-7/9 family outgroup*	JTT + G	JTT + G
*IL-4/13 family no outgroup*	WAG+G	WAG+G
*IL-4/13 family outgroup*	WAG+G	WAG+G
*IL-6 superfamily*	JTT + C10 + F	LG + F + G
*IL-6Rα family*	JTT + I + G	JTT + I + G
*IL-2Rα/IL-15Rα*	WAG+I + G	WAG+I + G
*Class-1 Group-2 cytokine receptors*	JTT + C30 + F	WAG+F + I + G
*ROR no outgroup*	JTT + I + G	JTT + I + G
*ROR HR3 outgroup*	JTT + I + G	JTT + I + G
*ROR RAR outgroup*	JTT + G	JTT + G
*FOXP no outgroup*	JTT + C20 + F	JTT + I + G
*FOXP invertebrate outgroup*	JTT + C10 + F	JTT + F + I + G

**Fig. 2:**
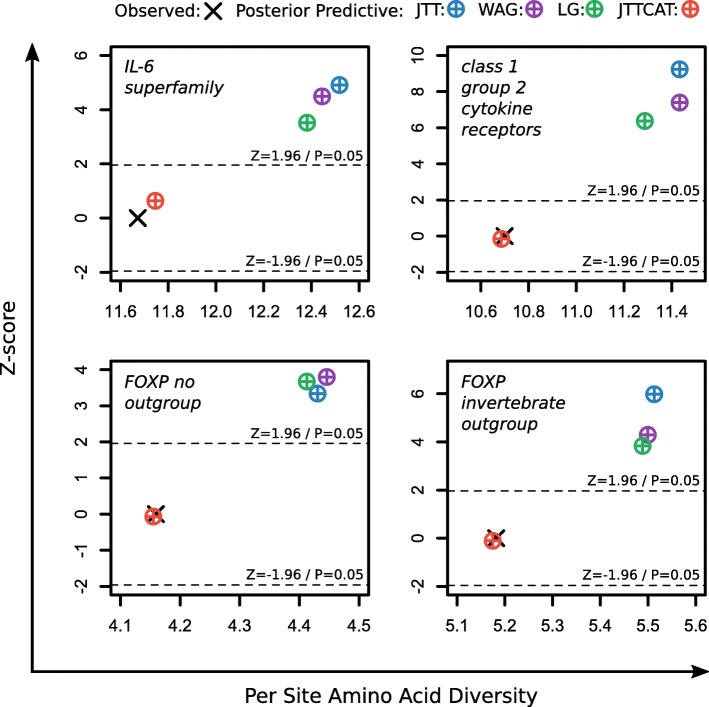
Posterior predictive simulations show that standard models, but not site-heterogeneous mixture models, inadequately capture site-specific biochemical diversity in all tested vertebrate immune genes

To better understand the above findings, we also compared the absolute fit of standard substitution models and mixture models using PPS (Fig. 2) [44]. Specifically, for each dataset better-fit by a paired standard and empirical CAT model, we tested the ability of standard models and non-empirical CAT-based models (this was JTT + CAT in all cases here) to anticipate evolutionary process variation across sites. PPS of the number of amino acids per site revealed that standard models failed to adequately capture site-wise biochemical diversity in all tested datasets, but instead consistently overestimated the per site amino acid alphabet (Z-score > 1.96; *P*-value < 0.05) (Fig. 2). This means that the ability of standard models to accurately infer homoplasy in these datasets is impaired, which could mislead phylogenetic inference and result in errors in branch lengths and evolutionary relationships [44]. On the other hand, CAT-based models adequately captured per-site amino acid alphabet diversity in every dataset (1.96 > Z-score > − 1.96; *P*-value > 0.05) (Fig. 2), and as such should be more robust to error for these datasets.

### Phylogenetic analyses of CD4+ T cell-associated genes

#### IL-2 superfamily

**Fig. 3:**
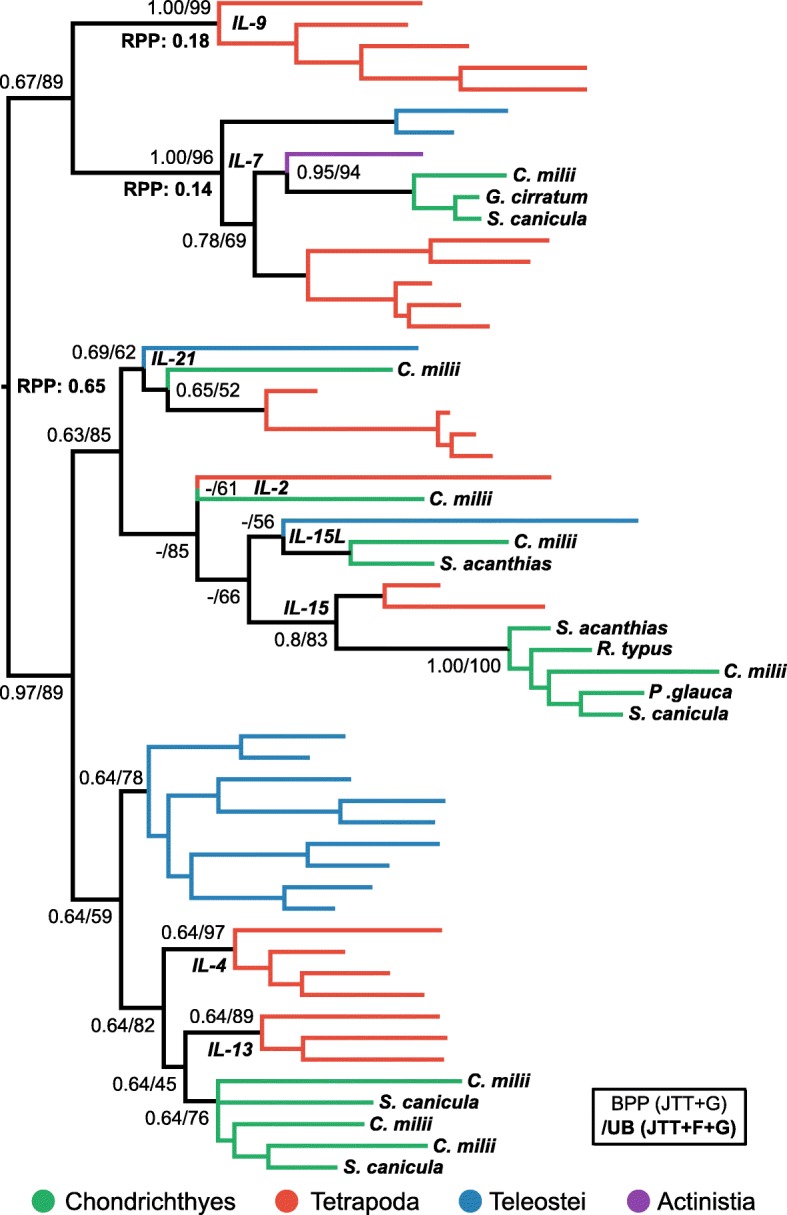
Phylogenetic analysis of the IL-2 superfamily. Branches are coloured according to the taxonomic key in the figure. Statistical support is shown for key nodes as per accompanying box, wherein the analysis shown in bold is the topology shown. Root Posterior Probabilities (RPP) > 0.05 from the BEAST analysis are shown

The IL-2 superfamily consists of three subfamilies, the IL-2 family, the IL-4/13 family, and the IL-7/9 family, and these were verified here by phylogenetic analysis (Fig. 3). Within the IL-2 subfamily there are two CD4+ T cell associated cytokines; IL-2 is a key inducer of T_reg_ cells, which modulate immune responses, promote feto-maternal tolerance, and help avoid allergy and autoimmunity [17], while IL-21 is the effector cytokine of T_FH_ cells, which contribute to antibody affinity maturation and immune memory [8]. Dijkstra [18] identified putative orthologues of mammalian IL-2 and IL-21 in elephant shark, despite initially being thought absent [5]. Our phylogenetic analyses of the IL-2 superfamily are consistent with these findings although support was weak (BEAST posterior probability [BPP] = 0.69 and ultrafast bootstrap [UB] = 62 for IL-21, and UB = 0.61 for IL-2) (Fig. 3). We also identified cartilaginous fish orthologues of IL-15 (BPP = 0.8; UB = 83), the other member of the IL-2 family, while the putative IL-21 identified in our homology searches turned out to instead group with the recently identified IL-15L (UB = 56) [94]. More focused analyses of the IL-2 family failed to provide improved phylogenetic resolution (results not shown), however this was not the case for the IL-7/9 and IL-4/13 families.

#### IL-7/9 family

**Fig. 4:**
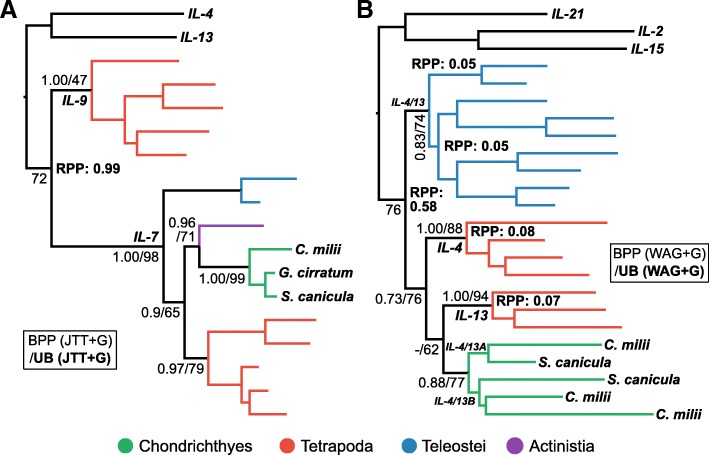
Phylogenetic analyses of the (**a**) IL-7/9 family and the (**b**) IL-4/IL-13 family. Outgroup sequences are from human. BPP and RPP values are from analyses not including the outgroups. Other details as per Fig. 3

IL-9 is the signature effector cytokine produced by mammalian T_H_9 cells, a subtype with a role in mucosal immunity that includes the expulsion of intestinal worms [9, 14]. IL-7 is considered the closest relative of IL-9 [65, 66], meaning that although we did not find a putative IL-9 orthologue in small-spotted catshark, the initial BLAST-based assignment of an IL-7 gene in elephant shark [5] implied either that IL-9 diverged from IL-7 after the divergence of cartilaginous fishes from other jawed vertebrates, or that IL-9 was lost or is yet to be found in cartilaginous fishes. To assess this, we performed phylogenetic analysis of the IL-7/9 family, using both outgroup and relaxed clock rooting approaches, to independently verify the root placement (root posterior probability [RPP] = 0.99). The putative IL-7 sequences from cartilaginous fishes form a clade with IL-7 from other vertebrates (BPP = 1.00; UB = 97%) (Fig. 4a). These results support a scenario where IL-7 and IL-9 both existed in the jawed vertebrate ancestor, with IL-9 subsequently being lost, or yet to be discovered, in cartilaginous fishes. This hypothesis is supported by the larger IL-2 superfamily analysis (Fig. 3).

#### IL-4/13 family

The IL-4/13 family of cytokines are the effector cytokines produced by T_H_2 cells in mammals, a subtype central to antibody-mediated immunity and the clearance of extracellular pathogens [95]. Evidence for putative IL-4/13-like sequences in elephant shark was provided by Dijkstra [18] after Venkatesh et al. [5] originally reported the absence of these genes from cartilaginous fishes. Our results indicate that two IL4/13-like lineages are present and expressed in small-spotted catshark (IL-4/13A and IL4/13B), having duplicated in the ancestor of cartilaginous fishes (BPP = 0.88; UB = 77%), but do not support clear orthology of these genes, or those of teleosts, to mammalian IL-4 or IL-13 (Fig. 4b). The IL-2 superfamily analysis was also congruent with these findings (Fig. 3).

#### IL-6 superfamily

**Fig. 5:**
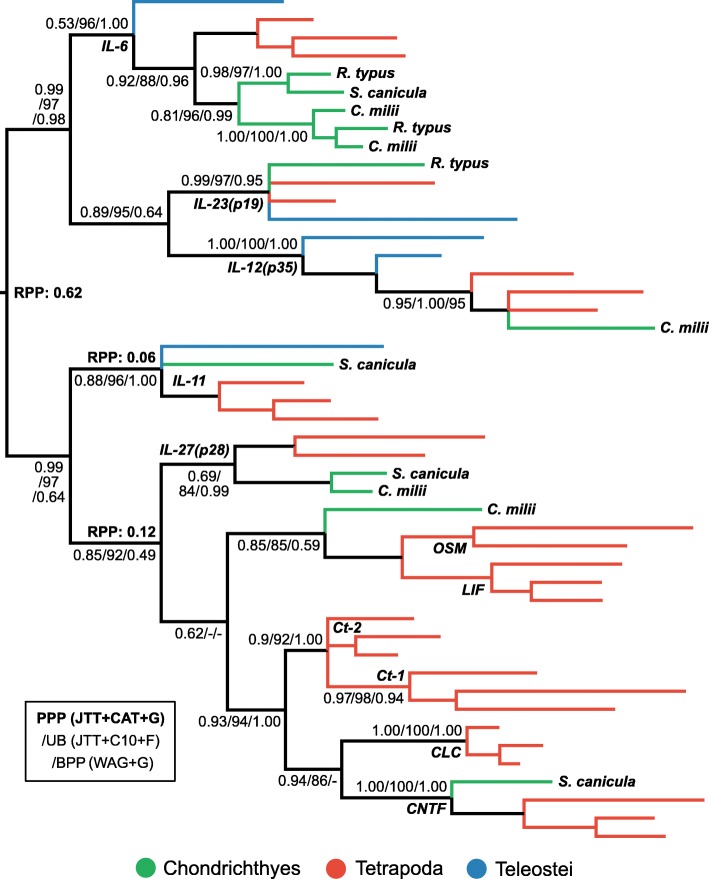
Phylogenetic analysis of the IL-6 superfamily reveals orthologues of IL-23α (p19), IL-27α (p28), and IL-11 in cartilaginous fishes. Details as per Fig. 3

IL-27α (p28), thought to be absent from cartilaginous fishes [5], forms half of the T_r_1 subset-inducing cytokine IL-27. T_r_1 cells dampen autoimmunity and inflammation by promoting expression of the immunosuppressive cytokine IL-10 [16]. IL-23 (p19), which is also thought not to exist in cartilaginous fishes [5], is a pro-inflammatory T_H_17 cell effector cytokine [10, 12]. Phylogenetic analyses of the IL-6 superfamily, to which these cytokines belong [62–64], reveal that the putative cartilaginous fish IL-27α sequence is sister to tetrapod IL-27α (BPP = 0.99; UB = 84%; PPP = 0.69), indicating orthology (Fig. 5). The phylogenetic analyses performed here are at odds with the prior assumption that IL-27 (p28), is closely related to pro-inflammatory IL-23 and the T_H_1-inducing cytokine IL-12 (p35) [96, 97]. Rather, IL-23 (p19), and IL-12 (p35) form a subfamily with the pro-inflammatory cytokine IL-6 (BPP = 0.99; UB = 97%; PPP = 0.98), and, depending upon root placement, IL-11, which modulates placentation, bone resorption, platelet production, as well as immune responses in mammals (Fig. 5). Phylogenetic analysis verified the identity of cartilaginous fish IL-23 (BPP = 0.95; UB = 97%; PPP = 0.97), as well as supporting the assignment of a cartilaginous fish IL-12 (p35)/IL-23 (p19) orthologue (BPP = 1.00; UB = 100; PPP = 1.00), indicating that these genes emerged in the jawed vertebrate ancestor (Fig. 5). Although IL-11 was not found in elephant shark by Venkatesh and colleagues [19], we found evidence of a cartilaginous fish orthologue which formed a clade with IL-11 of tetrapods (BPP = 1.00; UB = 96%; PPP = 0.88) (Fig. 5). Further, cartilaginous fish sequences form part of a clade with IL-6 from other vertebrates (BPP = 0.96; UB = 88%; PPP = 0.92), with evidence of lineage-specific duplication or triplication in cartilaginous fishes (Fig. 5). Beyond this, our analyses support the presence of orthologues to OSM/LIF (BPP = 0.59; UB = 85%; PPP = 0.85) and CNTF (BPP = 1.00; UB = 100%; PPP = 1.00) (Fig. 5). CNTF and CLC form a clade (UB = 86%; PPP = 0.94), which is sister to (BPP = 1.00; UB = 94%; PPP = 0.93) another clade containing Ct-1 and Ct-2 (BPP = 1.00; UB > = 92%; PPP = 0.9), suggesting that a CLC orthologue (and a Ct-1/Ct-2-like sequence) existed in the jawed vertebrate ancestor (Fig. 5).

#### IL-6Rα family

**Fig. 6:**
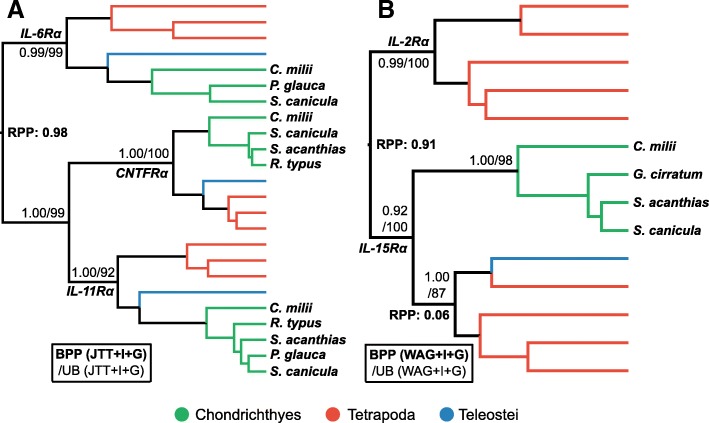
Phylogenetic analysis of the (**a**) IL-6Rα family, and the (**b**) IL2Rα/IL-15Rα family. Details as per Fig. 3

In mammals, IL-6Rα is expressed on both T_FH_ and T_H_17 subsets; T_FH_ cells are important for antibody production, affinity maturation of the antibody response, and memory B cell differentiation [8], while T_H_17 cells play a pro-inflammatory role, helping to maintain the integrity of mucosal barriers in humans [10]. For phylogenetic analyses involving putative cartilaginous fish IL-6Rα sequences, we included the closely related IL-11Rα and CNTFRα proteins [60], and employed a relaxed clock rooting approach [87]. The results firmly place the root between IL-6Rα and the other two proteins (RPP = 0.98), indicating that IL-11Rα and CNTFRα are more closely related to each other than to IL-6Rα (BPP = 1.00; UB = 99%) (Fig. 6a). Both Bayesian and maximum likelihood phylogenetic analyses strongly support direct orthology of cartilaginous fish IL-6Rα sequences to those in other jawed vertebrates (BPP = 0.99; UB = 99%) demonstrating that an *IL-6Rα* gene was present in the jawed vertebrate ancestor (Fig. 6a). Moreover, this approach also robustly supports the existence of cartilaginous fish orthologues of IL-11Rα (BPP = 1.00; UB = 92) and CNTFRα (BPP = 1.00; UB = 100) (Fig. 6a).

#### IL-2Rα/IL-15Rα family

IL-2Rα forms part of the IL-2R heterotrimer, which is pivotal to maintenance and growth of the immunomodulatory T_reg_ lineage [17], but is thought to be missing from cartilaginous fishes [5]. Dijkstra [18] suggested that IL-2Rα separated from IL-15Rα early in tetrapod evolution, and that IL-15Rα functionally accommodates the role(s) of IL-2Rα in teleost fishes. Our BLAST and HMMER searches identified putative orthologues of IL-15Rα, and while no appropriate outgroup is known, we performed relaxed clock rooted phylogenetic analyses of IL-2Rα and IL-15Rα. This result appears to verify the identity of cartilaginous fish IL-15Rα (BPP = 0.92; UB = 100) (Fig. 6b). Interestingly however, we found no evidence for IL-2Rα emerging from IL-15Rα, rather it seems that they diverged from a common ancestor prior to the divergence of cartilaginous fishes and bony vertebrates (RPP ≥ 0.97) (Fig. 6b).

#### IL-23R and the class 1 group 2 cytokine receptor family

**Fig. 7:**
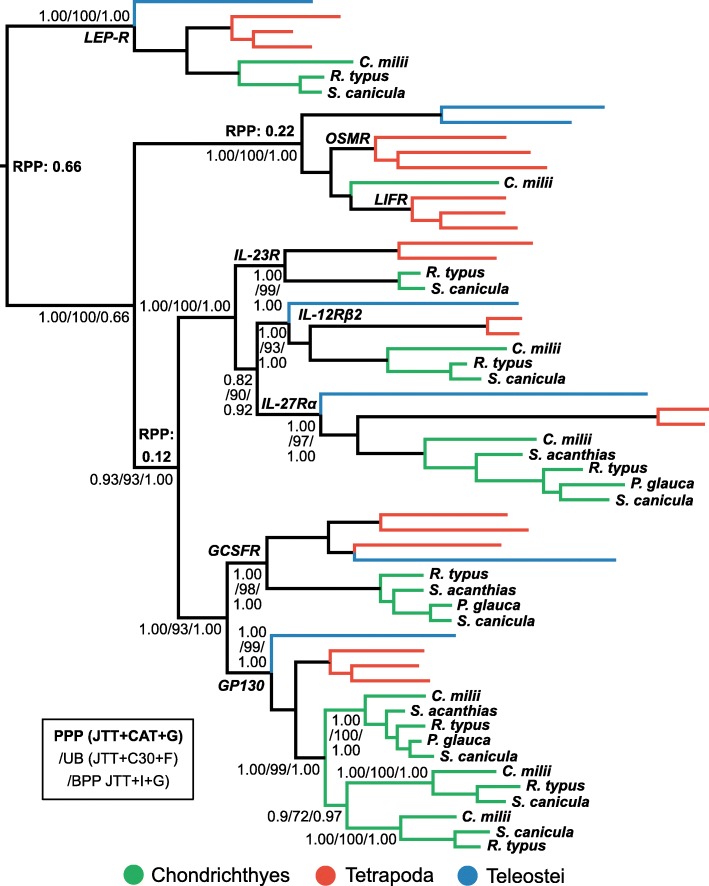
Phylogenetic analysis of class 1 group 2 cytokine receptors reveals an IL-23R orthologue in cartilaginous fishes. Details as per Fig. 3

IL-23R is a cytokine receptor specific to T_H_17 cells [8, 10, 12, 15]. To verify the putative IL-23R identified by BLAST in cartilaginous fishes, and to better understand the evolution of cytokine receptors, we carried out a phylogenetic analysis of the class 1 group 2 cytokine receptor family [61]. This revealed that putative cartilaginous fish IL-23R falls sister to IL-23R of tetrapods (BPP = 1.00; UB = 99%; PPP = 1.00) indicating the presence of an IL-23R orthologue in cartilaginous fishes (Fig. 7). The analyses support inclusion of IL-23R within a subfamily that also contains IL27Rα, and IL-12Rβ2 (BPP = 1.00; UB = 100; PPP = 1.00) (Fig. 7). IL27Rα and IL-12Rβ2 are involved in T_H_1 cell differentiation and, due to their relationships to bony vertebrate sequences, our data suggest that direct orthologues exist for these genes in cartilaginous fishes (BPP = 1.00; UB = 97%; PPP = 1.00, and BPP = 1.00; UB = 93%; PPP = 1.00, respectively) (Fig. 7).

This analysis also provides insights into the evolution of the other included class 1 group 2 cytokine receptors. GP-130 (also known as IL-6Rβ or IL-6ST), which forms complexes with many IL-6 and IL-6R family members, plays a key role in both promoting and suppressing inflammation, and is essential for embryo survival in mammals [98]. Three copies of GP-130 exist in cartilaginous fishes [5], which evidently result from two lineage-specific duplications (BPP ≥ 0.97; UB ≥ 72%; PPP ≥ 0.9) (Fig. 7). A GCSFR clade, which also contains cartilaginous fishes (BPP = 1.00; UB = 98%; PPP = 1.00), falls sister to GP-130 (BPP = 1.00; UB = 93%; PPP = 1.00), which together are sister to the IL-23Rα, IL-27Rα, and IL-12Rβ2 clade (BPP = 1.00; UB = 93%; PPP = 0.93) (Fig. 7). Outside this grouping, a cartilaginous fish sequence falls within an OSMR (multifunctional) and LIFR (tumour metastasis suppressor [99]) clade (BPP = 1.00; UB = 1.00%; PPP = 1.00) (Fig. 7). Finally, cartilaginous fishes possess a putative orthologue of leptin receptor (LEP-R), a hypothalamic appetite-controlling hormone receptor, as this sequence formed a clade with bony vertebrate LEP-R (BPP = 1.00; UB = 100%; PPP = 1.00), and relaxed clock rooting analysis best places the root between LEP-R and the other family members (RPP = 0.66) (Fig. 7).

#### ROR transcription factor family

**Fig. 8:**
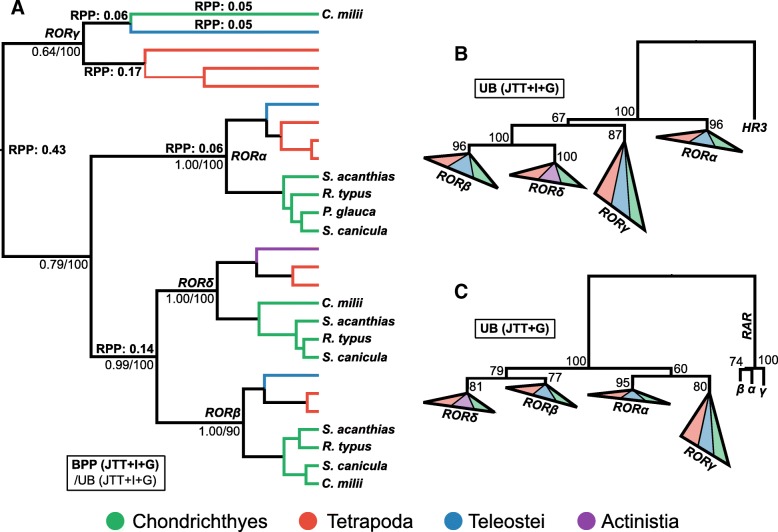
Phylogenetic analyses of the vertebrate ROR family shows that ROR-γ existed in the jawed vertebrate ancestor and reveals a new vertebrate ROR-β paralog not found in mammals (which we name ROR-δ). Alternative rooting strategies, using (**a**) a relaxed clock model, (**b**) fruit fly HR3 as outgroup, or (**c**) human RARs as outgroup, show that the root of the ROR phylogeny cannot be confidently placed. Gene level clades are collapsed in (**b**) and (**c**), but contain the same taxa as **a** Other details as per Fig. 3

Having identified orthologues of two cytokine receptors associated with the T_H_17 subset (IL-23R and IL-6R), we performed a variety of phylogenetic rooting analyses to look for evidence of the transcription factor ROR-γ, the master regulator of T_H_17 cells [100]. ROR-γ is a member of the larger ROR family and was reported missing in elephant shark [5]. We tested a relaxed clock rooting method (Fig. 8a), and two alternative outgroups; fruit fly HR3 (Fig. 8b), and the human RAR family (Fig. 8c), both closely related nuclear receptors [67]. These approaches did not provide congruent support for any root position (Fig. 8), which may result from the major difference in evolutionary rate between ROR-γ and the other RORs (Fig. 8). However, our results are consistent with two new findings: (*i*) ROR-γ existed in the jawed vertebrate ancestor, though evidence for its presence in cartilaginous fishes depends on root placement in relaxed clock analyses (Fig. 8a), and (*ii*) a fourth member of the vertebrate ROR family, which falls sister to ROR-β (BPP = 0.99; UB ≥ 79%), exists, but is possibly lost in mammals and teleosts. We propose the name ROR-δ for this new family member (Fig. 8).

#### FOXP transcription factor family

**Fig. 9:**
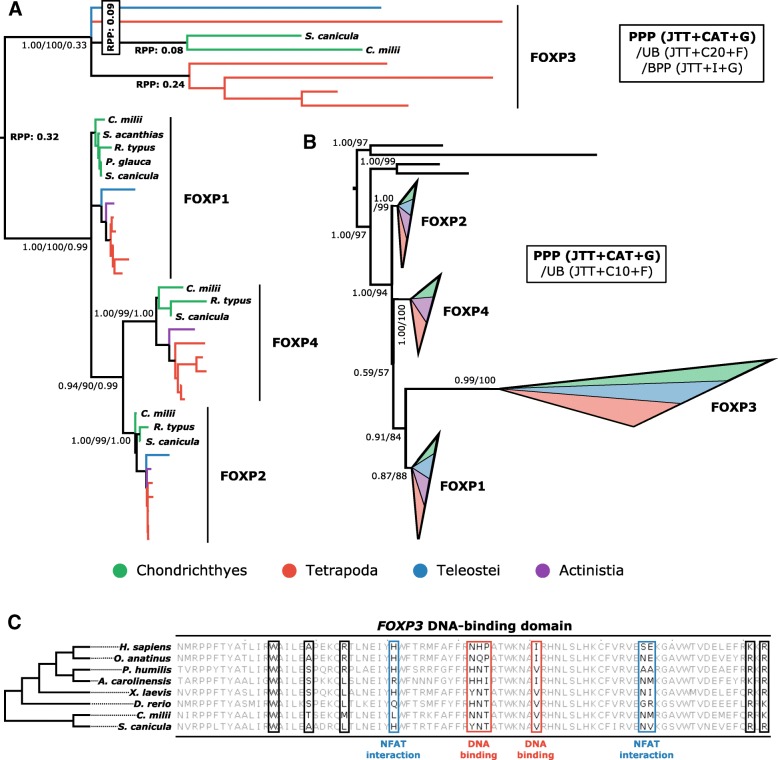
Phylogenetic analyses of the vertebrate FOXP family verifies the existence of cartilaginous fish orthologues to FOXP1–4, but alternative rooting strategies, using (**a**) a relaxed clock model, or (**b**) invertebrate FOXP sequences as an outgroup, show that the root of the FOXP phylogeny cannot be confidently placed. Other details for (**a**) and (**b**) as per Figs. 3 and 6. (**c**) Alignment of the FOXP3 DNA-binding domain from phylogenetically representative vertebrates suggests that cartilaginous fish FOXP3 is not atypical

FOXP3 is the master regulator of T_reg_ cell development and function in mammals [101]. A FOXP3 homologue was identified in elephant shark, but presumed non-functional by Venkatesh and colleagues [5] based on analysis of the DNA-binding domain. However, Dijkstra [18] has suggested that this inference may be premature. Our phylogenetic analyses suggest that cartilaginous fishes possess orthologues to all four mammalian FOXP family members (Fig. 9). Like the ROR family, the relationships between these genes are not easily resolved as different root positions are favored when the tree is rooted with either relaxed clocks or invertebrate FOXP sequences (Fig. 9). Another common feature between the FOXP and ROR families is a striking increase in evolutionary rate in the family member involved in T cell biology (i.e. immune functioning RORC and FOXP3), as compared to the other family members (Figs 8 and 9). We generated a multiple sequence alignment of cartilaginous fish FOXP3 DNA binding domains against those of other jawed vertebrates to explore the issue of FOXP3 functionality in cartilaginous fishes and the jawed vertebrate ancestor. This revealed that the sites predicted to lead to non-functionality in cartilaginous fishes by Venkatesh et al. [5] are not noticeably more divergent from human than those of other non-mammals, and certainly no more so than expected in the context of species phylogeny and divergence times [1, 2].

## Discussion

### Transcriptomes, taxonomy, and gene discovery

As available genomic data remains relatively sparse for cartilaginous fish, we generated a normalised multi-tissue transcriptome for the small-spotted catshark, with the goal of maximizing representation of novel transcripts. We applied a variety of trimming approaches and tested subsequent assemblies using various statistical approaches. While some of the assemblies contained excessive numbers of transcripts considering the number of genes typical of a vertebrate genome, we did not introduce filters by coverage, length, or contamination, thus retaining as many novel transcripts as possible. The results indicate that while statistical methods can be useful to determine the most contiguous (e.g. high N50), or most complete assembly (e.g. fewest missing BUSCOs), choosing a ‘best’ assembly may lead to loss of interesting data, such as novel sequences or full-length transcripts. The findings similarly highlighted the differential presence of transcripts of interest in our datasets compared to those of past transcriptome studies of the same species [6, 30], to the genome of the distantly related elephant shark [5], and to genomic data from other shark species. Our study thus supports the notion that using a single genome [102] or transcriptome assembly [103], or species [104], is grossly insufficient to adequately assess gene presence or absence in a vertebrate class. Our results also suggest that paired-end data, or longer reads than those applied here, should also be utilised where possible. Despite this, the data generated in this study contains novel sequences for cartilaginous fishes, and other researchers should benefit from this resource.

### Adequate phylogenetic modelling of fast-evolving immune genes

A precarious balance must be maintained in immune gene evolution to uphold structural integrity and functionality, while avoiding pathogen subversion. As such, immune genes evolve rapidly, but with strong site-specific evolutionary pressures; both of which can contribute to accumulation of hidden substitutions (homoplasy) over time, which is known to cause phylogenetic errors. In line with this, standard phylogenetic models inadequately predicted the diversity of amino acid alphabets across sites in the immune gene datasets tested in this study. This inadequacy to detect site-specific biochemical constraints indicates that a model has an impaired capacity to infer hidden substitutions in the data [44]. To the best of our knowledge this is the first report of the inadequacy of standard phylogenetic models for immune gene datasets, though this result is not surprising given the complex evolutionary pressures imposed on immune genes by the host-pathogen arms race. In stark contrast, and consistent with our hypothesis that site-heterogeneous models would better accommodate the rapid and complex evolutionary patterns of immune genes, CAT-based models adequately captured site-specific amino acid alphabet diversity for all tested datasets. These findings imply (based on [44]) that standard models will often fit poorly to immune gene datasets, and that CAT-based models should typically produce more accurate phylogenetic trees for immune genes in future studies.

### The problem of rooting rate asymmetric phylogenetic trees

Increased attention has been given recently to the prevalence of asymmetric evolutionary rates between different members of gene families and the negative impact this has on phylogenetic inference [105, 106]. Here, for the ROR and FOXP transcription factor family phylogenetic analyses we found that the immune genes RORC and FOXP3 had drastically increased evolutionary rates compared to their relatives. In the case of outgroup-free relaxed clock rooting analyses, the root fell between the fast-evolving immune gene and the rest of the family, although this was never the case using outgroups. This suggests that clock rooting may be susceptible to error in the face of extreme rate asymmetry, even when an uncorrelated relaxed clock model [87] is applied. However, multiple alternate outgroups were tested for the ROR family and these resulted in different root positions, meaning that the root placement under the relaxed clock cannot be reliably dismissed. Interestingly, it appears that for families of immune genes with a shared fast-evolutionary rate this phylogenetic difficulty is not as prevalent, with clocks and outgroups supporting a common root position (e.g. IL-6R family, IL-7/9 family, IL-4/13 family). As such, while many factors may contribute to the phylogenetic incongruence in the transcription factor families analysed here (e.g. rediploidisation following genome duplication events prior to divergence of cartilaginous and bony vertebrates [37, 39, 107], or selective pressure changes associated with the functional shift to immune gene status inducing compositional heterogeneity among branches, heterotachy, and/or heteropecilly [108]) we nonetheless predict that rate asymmetry is likely a key player, promoting the case for it being a somewhat overlooked phenomenon [105]. We suspect that this may derive from standard substitution models being designed to accommodate rate asymmetry, and the resultant branching errors when this fails being less obvious at the level of genes than species, where there are often morphology-derived topological expectations.

### CD4+ T cell subsets in cartilaginous fishes and the jawed vertebrate ancestor

**Fig. 10:**
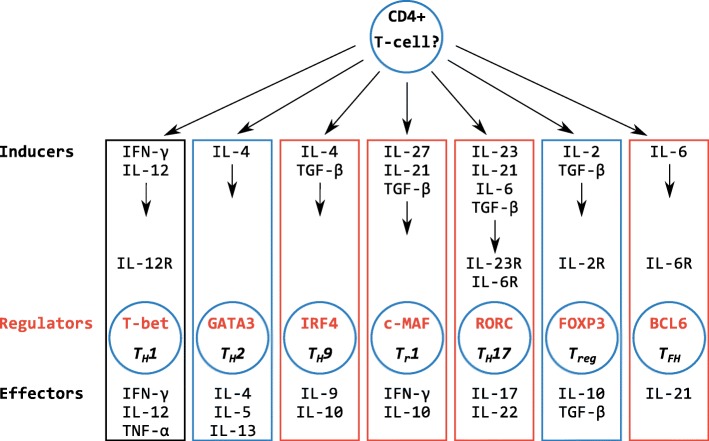
A full set of T helper and T regulatory cell associated genes existed in the jawed vertebrate ancestor. The figure and gene selection are based on Fig. 5 from Venkatesh et al. [5], but here refer to the ancestral jawed vertebrate gene set rather than that of cartilaginous fishes. Boxed lineages were predicted to have emerged in the ancestor of jawed vertebrates by Venkatesh et al. [5] (black boxed lineages), by this study (red boxed lineages), or by this study and Dijkstra [18] (blue boxed lineages). All genes listed, except for IL-9 and IL-2Rα, have now been identified in cartilaginous fishes

Venkatesh et al. proposed that cartilaginous fishes have only basic or primordial T cell function [5]. Here, having employed detailed phylogenetic analyses, we identified orthologues of several additional genes integral to CD4+ T cell-subset induction and function in cartilaginous fishes. Combined with previous findings [5, 18, 19], these results show that cartilaginous fishes possess the molecules necessary to generate an array of CD4+ helper and regulatory T cells comparable to that of mammals. In fact, we present a new model of helper and regulatory T cell evolution wherein all key genes (in some form) and/or pathways found in mammals existed in the jawed vertebrate ancestor (Fig. 10).

We have provided new insights on the controversy surrounding the absence of IL-2R and FOXP3 functioning in cartilaginous fishes, both of which are required for the development and function of T_reg_ cells, a subset that helps maintain self-tolerance by dampening inflammation and suppressing immune responses [12, 17]. For example, in teleost fishes a common IL-2/15 receptor binds both IL-2 and IL-15 [18, 94, 109]; in a similar manner IL-15R, which our study shows is present in cartilaginous fishes, could functionally compensate for the lack of IL-2R, which appears to be lost from both cartilaginous fishes and teleosts. Also, while cartilaginous fish FOXP3 shows poor conservation of the amino acids that facilitate DNA binding in mammalian FOXP3, we find that this is not unusual among non-mammals. Further, these residues vary naturally between FOXP subfamily members—all of which can bind DNA [110]—so lack of conservation of these elements in FOXP3 does not necessarily equate to an absence of T_reg_ cells in cartilaginous fishes [18]. Further, while Venkatesh et al. used the apparent absence of T helper cell subsets in general, and T_FH_ cells in particular, to explain the long lag-times associated with humoral immune responses in cartilaginous fishes [5], our results contradict this idea. Indeed, our data suggest cartilaginous fishes are capable of producing both T_H_2 and T_FH_ cells, a finding that fits better with the antibody affinity maturation and immunological memory previously evidenced in cartilaginous fishes [20, 21].

While our data is consistent with the presence of a sophisticated, mammalian-like, set of T cell subtypes in cartilaginous fishes, several lineage-specific novelties were also observed; for example, GP-130 (IL-6Rβ/IL-6ST; the signalling component of the IL-6 receptor) is triplicated in cartilaginous fishes, potentially increasing the diversity of signalling that can be induced by IL-6. In line with this, IL-6 is also duplicated (and possibly triplicated) in cartilaginous fishes. Enigmatic orthology, as observed for the IL-4/13 family, may result from independent duplications in many lineages, combined with exon shuffling or conversion events [19]. Lineage-specific loss events have also played a role, for example the potential loss of IL-9 and IL-2Rα in cartilaginous fishes, or ROR-δ in mammals.

Finally, it must be noted that the data presented here do not provide conclusive evidence for the existence of any T cell subset in cartilaginous fishes, or the jawed vertebrate ancestor, but do strongly reject past conclusions regarding their absence. Importantly, although a canonical (i.e. mammalian-like) CD4 was reported as absent from cartilaginous fishes [5, 19], one of several CD4/LAG3-like molecules identified by Venkatesh et al. [5] has since been shown to have a CD4-like expression profile and thus may act as the functional equivalent in sharks (Martin F. Flajnik, personal communication). Together with our data, this suggests that a fully developed set of CD4+ helper and regulatory T cell subsets equivalent to that of mammals evolved in the jawed vertebrate ancestor and still exists, with lineage-specific modifications, in cartilaginous fishes today. While more work is required to fully understand T cell biology in cartilaginous fishes, our results show that this arm of their adaptive immune system is likely no more ‘primordial’ than that of mammals.

## Abbreviations

BPP: BEAST Posterior Probability
IL: Interleukin
IL-R: Interleukin receptor
KEA: King et al.
MEA: Mulley et al.
PPP: Phylobayes Posterior Probability
RPP: Root Posterior Probability
UB: Ultrafast Bootstrap
